# The River past and God Forgotten

**Published:** 1919-04-26

**Authors:** 


### THE RIVER PAST AND GOD FORGOTTEN.

We have always thought the old saw more cynical and epigrammatic than generally applicable, but it does seem as if there was some danger of nurses being forgotten now the war is past. Munition workers, who have done very well, it is true, but who have been in no war danger, receive unemployment pay for six months. Army nurses, who have done equally well and have endured all the dangers and many of the miseries of war, receive a fortnight's notice. This is bad enough, but there are worse cases, and we feel sure that it is only necessary to draw attention to them in order to have them remedied.

The British Farmers' Hospital was situated during the war at Calais, and here the hospital was subjected every night to several air-raids. Think of it, you Londoners, who were scared out of London in thousands and tens of thousands by fewer air-raids in six months than the nurses at Calais endured in a week! And remember that some of them endured this heroically for four years! Think of this, and ask yourselves if it is right and just that their salaries should remain unpaid.

The British Farmers' Hospital is now transferred from the administration of the British Red Cross to that of the Belgian Army, and is in course of removal from Calais to Brussels. The Belgian Army does not seem to want it very much, and the removal is proceeding in a very leisurely manner. The removal is being carried out by the British farmers at very heavy expense, and the Belgian authorities do not appear very anxious to hasten it. Meantime, the nurses who were transferred with the hospital are kicking their heels in idleness in Brussels. They are idle, not by choice, but by compulsion, and would be glad to be at work again; but there is no work for them until the hospital is reconstituted. Meanwhile, they are receiving no pay. Their salaries for January were not paid until the end of March, and their salaries for February and March have not been paid yet. Even their demobilisation money, due on January 31, has not been paid; and the feelings of the nurses are not much solaced by the knowledge that both the medical staff and the orderlies have been paid.

The only solatium the nurses have received is permission to ride free of charge in the tramways. This is something, no doubt, but the privilege is a good deal discounted by the very audible comments of their Belgian fellow-travellers, who express loudly their very strong opinion that the English ought to pay. As soon as peace is signed even this small privilege will be withdrawn, and the twenty-two nurses attached to the hospital will have to stay indoors or walk to their destination, however far that may be, for most of them are entirely dependent on their salaries, and by this time are penniless.

This is not as it should be, and we commend the matter to the attention of the home authority of the British Farmers' Hospital, an institution that has done splendid work during the war.

